# A Brief Review of Recent Theoretical Advances in Fe-Based Catalysts for CO_2_ Hydrogenation

**DOI:** 10.3390/molecules29061194

**Published:** 2024-03-07

**Authors:** Haoxiang Tang, Tongyue Qiu, Xuerui Wang, Chundong Zhang, Zunmin Zhang

**Affiliations:** State Key Laboratory of Materials-Oriented Chemical Engineering, College of Chemical Engineering, Nanjing Tech University, Nanjing 211816, China; tonghx@njtech.edu.cn (H.T.); tongyue@njtech.edu.cn (T.Q.); x.wang@njtech.edu.cn (X.W.)

**Keywords:** CO_2_ hydrogenation, Fe-based catalysts, density functional theory, reverse water–gas shift

## Abstract

Catalytic hydrogenation presents a promising approach for converting CO_2_ into valuable chemicals and fuels, crucial for climate change mitigation. Iron-based catalysts have emerged as key contributors, particularly in driving the reverse water–gas shift and Fischer–Tropsch synthesis reactions. Recent research has focused on enhancing the efficiency and selectivity of these catalysts by incorporating alkali metal promoters or transition metal dopants, enabling precise adjustments to their composition and properties. This review synthesizes recent theoretical advancements in CO_2_ hydrogenation with iron-based catalysts, employing density functional theory and microkinetic modeling. By elucidating the underlying mechanisms involving metallic iron, iron oxides, and iron carbides, we address current challenges and provide insights for future sustainable CO_2_ hydrogenation developments.

## 1. Introduction

Fossil fuels, including coal, oil, and natural gas, have historically served as the primary sources of energy to meet increasing global demands. However, their combustion releases substantial amounts of greenhouse gases, notably carbon dioxide (CO_2_), significantly contributing to climate change [1,2,3]. Efforts to develop efficient carbon dioxide capture and utilization technologies arise not only from the need to mitigate atmospheric CO_2_ levels but also from the opportunity to convert captured CO_2_ into valuable products [4,5]. Among these technologies, catalytic CO_2_ hydrogenation emerges as a highly promising approach for CO_2_ recycling, particularly when coupled with renewable hydrogen sources like solar, wind, or hydropower [6,7]. This approach’s attractiveness lies in its ability to utilize CO_2_ as a resource, transforming it into valuable chemicals such as methanol, olefins, and paraffin [8], thereby fostering sustainability and reducing reliance on fossil fuels [9]. As renewable energy technologies advance, catalytic CO_2_ hydrogenation is expected to play a pivotal role in carbon cycling and carbon neutrality initiatives [10]. Despite the substantial promise of catalytic CO_2_ hydrogenation, its broad adoption remains constrained by certain barriers [11]. Researchers are currently involved in actively optimizing catalyst design to improve reaction rates, selectivity, and stability in the hydrogenation process, utilizing both experimental and theoretical methods [12].

Fe-based catalysts are distinguished among metal catalysts by their demonstrated cost-effectiveness, abundant product yields, and exceptional catalytic performance in CO_2_ hydrogenation, encompassing both the endothermic Reverse Water–Gas Shift (RWGS) and exothermic Fischer–Tropsch Synthesis (FTS) processes [13,14,15]. Their proven activity in RWGS and FTS reactions has attracted attention for direct CO_2_ hydrogenation [16]. For example, iron oxide-based catalysts have exhibited notable efficiency in high-temperature water–gas shift reactions [17,18], while iron carbides (FeC_x_), particularly Hägg-carbide (χ-Fe_5_C_2_), are usually considered as the catalytically active phases in CO_2_-FTS [19,20,21]. Compared to Co-based and Ru-based catalysts, iron-based catalysts exhibit higher affinity for olefins and long-chain hydrocarbons [22]. Recent research has focused on alkali metal-modified Fe-based catalysts, such as sodium (Na), potassium (K), and rubidium (Rb) [23]. These modifiers have demonstrated the capacity to reshape the electronic structure of catalyst interfaces, leading to enhanced CO_2_ adsorption, the formation of Fe_5_C_2_-carbonates, and reduced energy barriers for olefin desorption [24,25,26]. Additionally, doping with transition metals like zinc (Zn), copper (Cu), manganese (Mn), and cobalt (Co) has refined catalyst evolution and reliability [24,27,28,29]. The interplay between different metals and oxygen adsorbed on iron species significantly influences dissociation activation energy and CO_2_ conversion pathways. In addition, zeolite-confined Fe-based catalysts have also garnered significant interest for CO_2_ hydrogenation, owing to their exceptional efficiency, versatile framework, and customizable composition. The role of zeolite properties, including topology, the nature, density, and strength of acid sites, and metal–zeolite interactions, has been comprehensively reviewed in the literature for its influence on catalytic activity and product selectivity [30,31,32].

In the field of catalysis research, quantum mechanics methods, particularly density functional theory (DFT), have become indispensable in recent decades [33,34,35]. These computational tools offer invaluable insights into the intricate atomic-scale mechanisms underlying CO_2_ hydrogenation. DFT has exhibited remarkable efficacy in specific aspects of CO_2_ hydrogenation, enabling the exploration of novel intermediates, the scrutiny of potential catalysts, and the elucidation of reaction pathways [36,37]. Moreover, the integration of experimental data with theoretical findings establishes crucial connections between catalytic activity and characteristics in RWGS, facilitating the advancement of cutting-edge catalytic materials [38,39]. Notably, DFT calculations of CO_2_ behavior on Fe-based catalyst surfaces provide crucial insights into adsorbed species evolution, offer information about rate-determining steps, and reveal overall reaction mechanisms [40,41]. This computational paradigm not only accelerates catalyst development for CO_2_ hydrogenation but also improves our understanding of chemical reactivity and the simulation of intricate catalytic reaction pathways [42,43].

While some previous studies have offered general or specific summaries of CO_2_ hydrogenation with iron-based catalysts [12,22,23,42,44], this review exclusively focuses on recent theoretical achievements in this field, predominantly employing density functional theory and microkinetic modeling. The principal objective is to elucidate the underlying mechanisms involving metallic iron, iron oxides, iron carbides, and the influence of alkali or transition metals on CO_2_ redox and association processes. This work aims to provide a comprehensive summary of the oxidation-reduction mechanism, as well as the associative pathways of CO_2_. Additionally, we anticipate the potential contribution of machine learning and artificial intelligence in driving novel advancements in catalyst design for CO_2_ hydrogenation.

## 2. Related Reactions and Computational Methods

**Figure 1:**
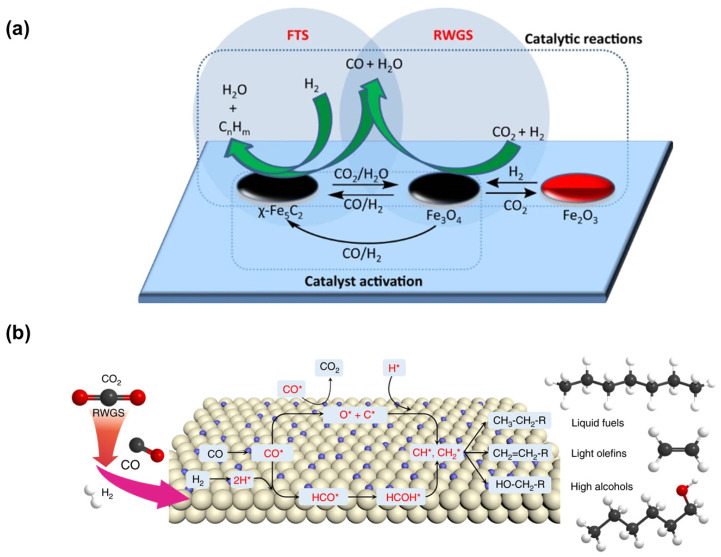
(**a**) Reaction scheme for CO_2_ hydrogenation to jet fuel range hydrocarbons. The CO_2_ hydrogenation to jet fuel range hydrocarbons process through a Tandem Mechanism in which the reverse water–gas shift reaction (RWGS) and Fischer–Tropsch synthesis (FTS) reaction are catalysed by Fe_3_O_4_ and χ-Fe_5_C_2_, respectively. Reprinted with permission from Yao et al. [48]. Copyright 2020 Springer Nature. (**b**) Scheme of CO_2_ modified FTS-based catalytic mechanism. Reprinted with permission from Ye et al. [51]. Copyright 2019 Springer Nature.

In the RWGS reaction, renewable hydrogen (H_2_) and CO_2_ undergo a redox reaction to generate carbon monoxide (CO) and water (H_2_O) (Equation (1)). Potential pathways include the formation of methane (CH_4_) (Equation (2)) and methanol (CH_3_OH) (Equation (3)) [45]. Governed by Le Chatelier’s principle, the endothermic nature of the reaction thermodynamically favors higher temperatures. Consequently, as temperatures decrease, the equilibrium gradually shifts to promote the exothermic reverse of Equation (1) and methanation reactions, which predominantly occur as side reactions [46]. Effective catalyst surfaces should facilitate CO desorption while impeding further hydrogenation into hydrocarbons, necessitating the precise design of catalysts and careful regulation of reaction conditions to optimize CO_2_ valorization. In contrast to the indirect route, where CO_2_ transforms into more active intermediates like CO or MeOH before conversion into hydrocarbons, the direct RWGS reaction is often coupled with FTS for the production of fuel range hydrocarbons from syngas (Figure 1a). In this process, Fe_3_O_4_ serves as a RWGS catalyst, while χ-Fe_5_C_2_ is employed for FTS [47,48]. This tandem CO_2_-FTS integrates RWGS for CO_2_ activation with C-C coupling through FTS, thus facilitating the generation of extended hydrocarbon chains (Figure 1b) [49,50,51].

CO_2_ + H_2_ ⇆ CO + H_2_O Δ*H* = 42.1 kJ/mol (1)

CO_2_ + 4H_2_ → CH_4_ + 2H_2_O Δ*H* = −165 kJ/mol (2)

CO_2_ + 3H_2_ → CH_3_OH + H_2_O Δ*H* = −49.5 kJ/mol (3)

The reaction kinetics data indicate that the main pathways for the RWGS reaction are the redox and association mechanisms [44,52,53], as presented in Table 1. In the redox mechanism, the catalyst oxidizes CO_2_ to form CO (CO_2_ → CO_2_* → CO* → CO). In the association mechanism, CO_2_ typically adsorbs onto the catalyst surface, leading to the formation of intermediate species such as COOH and HCOO during the reaction, which subsequently decompose into CO and H_2_O. COOH and HCOO pathways depend on CO_2_* and H* (CO_2_ → CO_2_* → COOH* → CO* → CO, CO_2_ → CO_2_* → HCOO* → HCO* → CO* → CO). The final products and the related multiphase catalytic processed involving CO_2_ intermediates and reaction surfaces are mainly affected by the reaction operating conditions and the composition of the Fe-based catalyst.

While catalyst development traditionally relies on trial-and-error experimental methods, the emergence of multiscale models for designing and refining catalysts has become indispensable [54]. Typically, scientists employ DFT to validate experimental findings, including catalytic conversion, selectivity, and characterizations [55]. The generalized gradient approximation (GGA) with the Perdew–Burke–Ernzerhof (PBE) functional for electron exchange–correlation energy approximation is commonly used for the structural optimization of Fe-based catalysts [56]. For transition metal oxides, conventional treatments often underestimate equilibrium lattice constants and overestimate binding energies, necessitating the incorporation of an effective Hubbard U value for accurate predictions [57]. Furthermore, conventional semi-local DFT-GGA functionals lack inherent inclusion of the long-range electronic correlations responsible for van der Waals (vdW) interactions. Consequently, there is a tendency to underestimate the binding affinity of sizable, closed-shell adsorbates interacting predominantly with catalytic surfaces via vdW interactions, such as aromatic compounds and extended hydrocarbon chains [58]. In heterogeneous catalysis, empirical vdW corrections are commonly used in DFT studies to address vdW interactions, rather than relying solely on vdW functionals [59]. Electronic structure calculations entail constructing an atomic-scale structural catalyst model and scrutinizing energy variations associated with fundamental steps in the reaction mechanism [60].

**Figure 2:**
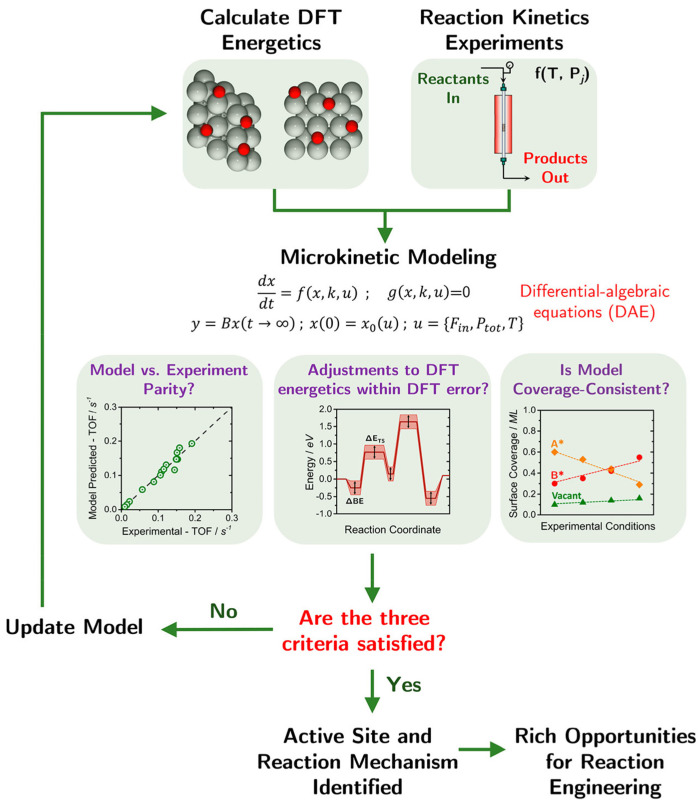
Proposed algorithmic scheme for elucidating the nature of the catalytic active site and the reaction mechanism, using a combination of DFT, reaction kinetics experiments, and microkinetic modeling. Reprinted with permission from Bhandari et al. [63]. Copyright 2020 American Chemical Society.

Despite the complexity of real catalyst materials, first-principle methods are employed to model chemical reactions at the atomic level, enabling the assessment of reaction properties and catalyst composition [61,62]. Microkinetic models (MKMs) are commonly utilized to unveil the fundamental reaction mechanisms of heterogeneous catalytic reactions, leveraging energy insights obtained from DFT calculations. Unlike electronic structure methods that elucidate how intermediates move on catalytic surfaces, MKMs bridge the gap between atomic-scale phenomena and macroscopic properties, facilitating the prediction of macroscopic reaction kinetics data. To precisely characterize the interactions between adsorbates and reaction energetics, Bhandari et al. [63] proposed an algorithmic framework that integrates first-principles calculations, microkinetic modeling, and reaction kinetics experiments (Figure 2). While the multiscale methodology remains the preferred technique for modeling heterogeneous catalysis, theoretical progress has enhanced the faithfulness and precision of each phase of the multiscale procedure [64]. The remarkable power of computational techniques to investigate the atomic-level intricacies of catalytic systems offers tremendous opportunities for the fundamental, bottom-up design of new heterogeneous catalysts [65].

## 3. Modelling Fe-Based Catalyst Surfaces

The heterogeneous catalytic conversion of CO_2_ and H_2_ occurs across a diverse range of iron-based catalysts, highlighting the need for a comprehensive understanding of CO_2_ activation mechanisms at their active sites. This demands thorough analysis, bridging diverse mechanisms with atomic-scale properties [66,67]. Numerous DFT studies have investigated CO_2_ adsorption on specific metal surfaces and H_2_ dissociation to elucidate conversion mechanisms [68,69]. However, a cohesive and accessible synthesis of these findings is still lacking [70]. Thus, this section aims to systematically summarize recent theoretical research on iron-based catalytic surfaces for CO_2_ hydrogenation and subsequent conversion into value-added products.

### 3.1. Iron-Based Catalysts

In FTS, the active phase of Co-based catalysts primarily exists in the metallic form, whereas Fe-based catalysts typically comprise complex species, including iron carbides, oxides, or metallic iron [71,72]. Iron species demonstrate the ability to produce both long-chain and short-chain hydrocarbons, with variations in product selectivity potentially attributed to iron’s comparatively weaker hydrogenation capability [73]. Nonetheless, the intricate interplay among these species considerably complicates the elucidation of their individual contributions to the overall reaction.

#### 3.1.1. Metallic Irons

**Figure 3:**
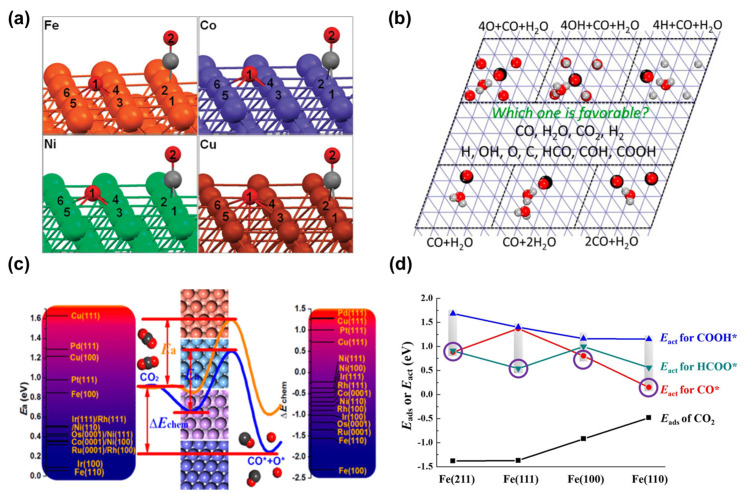
(**a**) The most stable calculated structures of (CO + O)/M(100) (M = Fe, Co, Ni, Cu). Reprinted with permission from Liu et al. [74]. Copyright 2012 American Chemical Society. (**b**) Adsorption of CO, CO_2_, HCO, COH, COOH, and HCOO on Fe (110). Adapted with permission from Liu et al. [75]. Copyright 2015 American Chemical Society. (**c**) CO_2_ adsorption and dissociation on low index surfaces of different transition metals. Reprinted with permission from Liu et al. [76]. Copyright 2018 American Chemical Society. (**d**) CO_2_ adsorption energy, dissociation and hydrogenation barriers on the various Fe surfaces. Reprinted with permission from Wang et al. [77]. Copyright 2018 Elsevier.

Significant scientific attention has been devoted to studying the intrinsic catalytic properties of CO_2_ adsorption and activation on Fe (100) and (110) surfaces. Liu et al. [74] employed DFT to explore the CO_2_ reduction process to CO on Fe, Co, Ni, and Cu surfaces. Their investigation highlighted the intrinsic chemical adsorption of CO_2_ on these surfaces (Figure 3a), driven by thermodynamic attributes. Notably, Fe (100) exhibited superior propensity for CO_2_ adsorption compared to the other metals studied. Valence electron density calculations provide insight into the CO_2_ activation, revealing charge transfer from the metal surface to the CO_2_ component. Overall, the total reaction energy barriers aligned with the trend of reaction energy: Fe < Co < Ni < Cu.

To evaluate the potential of metallic iron as a catalyst for the water–gas shift reaction, Liu et al. [75] meticulously simulated the interactions of CO and H_2_O on the Fe (110) surface at varying ratios (Figure 3b). They also investigated the effects of surface precoverage with 0.25 monolayer O, OH, and H. Their findings indicated that Fe (110) favored CO_2_ dissociation in terms of both kinetics and thermodynamics. Chen et al. [78] explored H_2_ activation and CO_2_ pre-hydrogenation reactions on four transition metal surfaces: Fe (111), Ni (111), Ru (111), and Pt (111). They discovered that the active hydrogen generated from H_2_ dissociation can influence the hydrogenation pathway of CO_2_. The metal electronegativity affects the selective hydrogenation of CO_2_. Specifically, on Pt (111) surface, active hydrogen can provide electrons through the Pt d_xy_ orbital, generating positively charged H^+^ species and favoring O binding of CO_2_. Conversely, on Fe (111) surface, active hydrogen accepts electrons via electron back-donation from the Fe d_xy_ orbital, leading to the formation of negatively charged H^−^ species, thereby facilitating the hydrogenation of the C-terminal of CO_2_. In another study, Liu et al. [76] systematically investigated the adsorption and dissociation of CO_2_ on various low-index transition metal surfaces (Figure 3c). They observed CO_2_ chemisorbing with a bent configuration on Fe (110). Moreover, they noted that CO_2_ activation is influenced by the type of transition metal and the surface geometry, with changes in charge transfer correlating with variations in adsorption energy.

Additionally, detailed investigations into the reaction mechanisms of CO_2_ and H_2_ on various iron surfaces, including changes in reaction energetics for intermediate formation, have been reported. Wang et al. [77,79] conducted comprehensive research exploring the adsorption, dissociation, and hydrogenation mechanisms of CO_2_ on Fe (100), (110), (111), and (211) surfaces (Figure 3d). They identified optimal configurations for CO_2_ and H_2_ on the Fe (100) surface, achieving enhanced adsorption and reactant activation through appropriate adjustment of the CO_2_/H_2_ ratio. Their research revealed that CO_2_ adsorption was more favorable on Fe (111) and (211) surfaces, while it was weakest on the (110) surface. Due to lower dissociation barriers, H_2_ on the Fe surface undergoes rapid activation and dissociation, emphasizing the significance of maintaining co-adsorption equilibrium of H_2_-CO_2_ for effective reactant activation. On the (100) and (110) surfaces, CO_2_ selectively undergoes direct dissociation to form CO*, while Fe (111) is more favorable for HCOO* formation owing to a lower kinetic barrier. However, the (211) surface shows a stronger competition between CO* and HCOO* formation. Notably, none of the Fe surfaces are conducive to COOH* intermediate formation. It is speculated that Fe (111) demonstrates excellent activation ability in CO_2_ conversion due to its lower initial hydrogenation barrier.

#### 3.1.2. Iron Oxides

**Figure 4:**
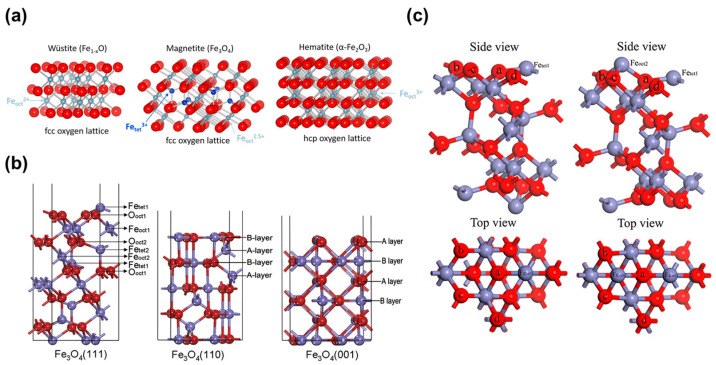
(**a**) The iron oxides are based on a close packed O^2−^ anion lattice with metal cations in octahedral and tetrahedral coordinated interstitial sites. Reprinted with permission from Parkinson [83]. Copyright 2016 Elsevier. (**b**) Fe_3_O_4_ (111), (110), and (001) surface and its different terminations (red for O; blue for Fe). Reprinted with permission from Yu et al. [88]. Copyright 2012 Elsevier. (**c**) Side view and top view for the Fe_tet1_-terminated and Fe_oct2-tet1_-terminated Fe_3_O_4_ (111) surfaces (blue, Fe atom; red, oxygen atom). Reprinted with permission from Su et al. [90]. Copyright 2016 Elsevier.

Fe catalysts characterized in situ and after the reaction have been identified in their oxidized states [12,52,80,81,82]. Parkinson [83] conducted a systematic review of various iron oxides’ surfaces, including magnetite (Fe_3_O_4_), maghemite (γ-Fe_2_O_3_), hematite (α-Fe_2_O_3_), and wustite (Fe_1−x_O) (Figure 4a). The discussion explored the correlation between the adsorption of molecules on the surface and the reactions of iron oxide catalysts, with a particular emphasis on the extraordinary thermal stability mechanism of isolated metal adatoms on the Fe_3_O_4_ surface. Chou et al. [84] demonstrated the promising activity of an unsupported Fe_3_O_4_ catalyst for the oxidative-reductive conversion of CO_2_ to form CO. Fishman et al. [85] focused on hematite nanosheets, nanowires, and nanoparticles to explore the nano-dimensionality of iron oxide based catalysis on the RWGS reaction. They investigated the mechanism by which iron oxide nanomaterials convert CO_2_ to CO through H_2_-TPR and RWGS and compared their findings with DFT-based H_2_ binding energy results. Band gap and reaction activity exhibit a certain correlation, with nanowires and nanosheets having lower band gaps being more active at low temperatures compared to nanoparticles with higher band gaps. The results showed that nanosheets exhibited a higher CO_2_ conversion rate of 28% at 510 °C, while nanowires achieved a remarkable conversion rate of 50% at 750 °C under atmospheric pressure.

In theoretical terms, Fe_3_O_4_ (111) emerges as the energetically favored surface [86]. Yang et al. [87] reported a systematic DFT study on the hydrogen adsorption behavior on the Fe_3_O_4_ (111) surface with two distinct terminal configurations, Fe_tet1_- and Fe_oct2_-termination. The findings revealed that on the Fe_tet1_-termination surface, H tends to adsorb and dissociate more readily, which is contrary to the adsorption behavior of CO on the same surface. Yu et al. [88] employed the GGA+U method to compute the properties of different Fe_3_O_4_ (111), (110), and (001) surfaces (Figure 4b). They utilized the GGA+U method to accurately capture the strong correlation effects of iron’s 3d orbitals and investigated the electronic structure, stability, and magnetism of Fe_3_O_4_. The computational results closely aligned with experimental lattice parameters and magnetic moments. Their study revealed that Fe_tet1_ and Fe_oct2_ are the most stable among the six Fe_3_O_4_ (111) terminations (O_oct2_, O_oct1_, Fe_oct1_, and Fe_tet2_), exhibiting similar surface energies. David et al. [89] performed the GGA+U approximation of DFT to model three different orientations of the Fe_3_O_4_ bulk. They investigated the stability of non-dipolar stoichiometric surface terminations and explored the redox properties of the surfaces. By introducing oxygen vacancies or adding oxygen atoms to the most stable non-dipolar stoichiometric surface terminations, they altered the redox conditions. Consistent with previous experimental STM images, the study concluded that (001) and (111) are the most stable surfaces of Fe_3_O_4_. While both (001) and (111) surfaces undergo oxidation under ambient conditions, they both experience a gradual reduction process. On the (001) surface, the reduction process initiates at lower chemical potentials and encompasses the stoichiometric plane. Su et al. [90] conducted first-principles calculations on the chemical adsorption of CO_2_ on Fe_oct2-tet1_- or Fe_tet1_-terminated Fe_3_O_4_ (111) surfaces (Figure 4c). The study revealed that the Fe_oct2-tet1_-terminated surface is more active compared to the Fe_tet1_-terminated surface. On the Fe_oct2-tet1_-terminated surface, CO_2_ acts as an electron acceptor from the surface, leading to the formation of covalent bonds between the C and O atoms of CO_2_ and the surface. This observation is supported by the analysis of the electron localization function, the difference electron density, and the partial density of states (PDOS). Han et al. [52] combined DFT and microkinetic analysis, utilizing CO_2_ and OH binding energies as descriptors, to investigate the CO_2_ hydrogenation reactions on typical iron-based surfaces (Fe_3_C, Fe_5_C_2_, Fe_2_O_3_, Fe_3_O_4_). In this theoretical framework, Fe_3_O_4_ is considered the predominant active phase for the RWGS reaction, and the direct CO_2_ dissociation mechanism governs the RWGS process. A degree of rate control analysis reveals that OH* removal is the rate-determining step for most iron-based surfaces [91,92,93]. By incorporating Cu and Zn into the Fe_3_O_4_ surface structure, the reaction can be effectively promoted. The authors believe that identifying the active phases before screening promoters is beneficial for analyzing multiphase catalysts with various surfaces.

#### 3.1.3. Iron Carbides

**Figure 5:**
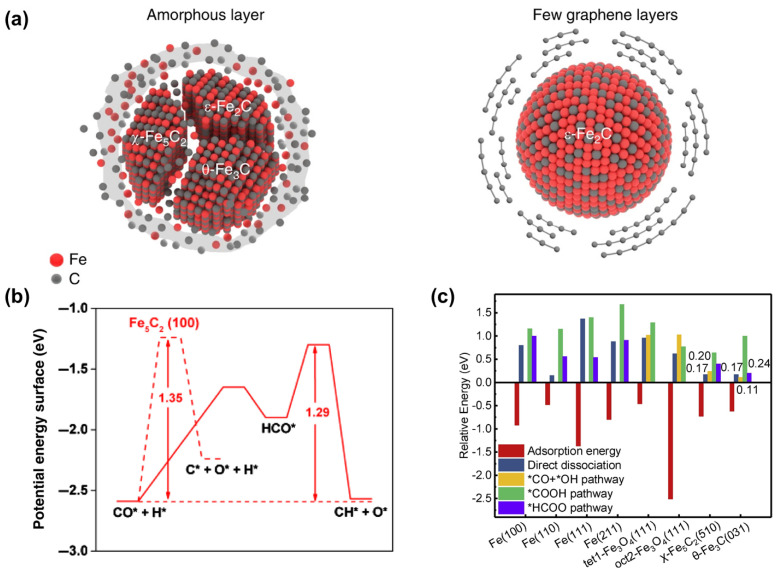
(**a**) Schematic models of iron-based catalysts for Fischer–Tropsch synthesis. Conventional catalysts with unconfined iron carbide (Fe_x_C) particles as the active phase. Graphene layer-confined ε-Fe_2_C. Reprinted with permission from Lyu et al. [95]. Copyright 2020 Springer Nature. (**b**) The potential energy diagrams for CO dissociation on Fe_5_C_2_ (100) (red) surface. The solid and dashed lines present direct and H-assisted CO activation pathways, respectively. The apparent activation barriers (in eV) are indicated. Reprinted with permission from Zhao et al. [96]. Copyright 2020 Chinese Chemical Society. (**c**) CO_2_ adsorption and activation energies on metallic iron, magnetite and iron carbides surfaces. Reprinted with permission from Liu et al. [97]. Copyright 2020 Elsevier.

In the study of iron carbides, researchers have focused on identifying the most stable active phases. Smit et al. [94] conducted a thorough analysis, integrating experimental and theoretical approaches to compare the reactivity of different ε-χ-θ carbide phases under varying conditions. The carbon chemical potential (μ_C_) was used as an indicator to describe thermodynamically induced phase transitions in the catalyst system. At low temperatures and high μ_C_ regimes, ε-carbide is primarily formed, whereas at lower μ_C_ conditions, the emergence of θ-Fe_3_C is observed. Under high-pressure FTS conditions, a gradual evolution from θ-Fe_3_C to χ-Fe_5_C_2_ was found. Additionally, the presence of an amorphous carbon/carbide layer commonly found on Fe-based catalysts tends to catalyze the transformation of carbides during FTS. Lyu et al. [95] proposed a novel catalytic approach by confining pure ε-Fe_2_C nanocrystals within a graphene matrix, resulting in enhanced activity and stability in contrast to traditional carbon-loaded Fe catalysts (Figure 5a). Additionally, DFT analysis further elucidates the interfacial interactions within the ε-Fe_2_C@graphene composite, demonstrating the efficacious suppression of amorphous carbon layer formation. Zhao et al. [96] synthesized a series of iron carbide (Fe_3_C, Fe_7_C_3_, and Fe_5_C_2_) nanoparticles, investigating their catalytic activity in FTS. They discovered that Fe_5_C_2_ exhibited superior catalytic activity and stability compared to other carbide phases (Figure 5b). DFT calculations further revealed that due to strong interactions with the dissociated atomic carbon on iron, iron carbides are inherently more active than elemental iron in terms of C-C coupling and methane formation. Moreover, the study demonstrated that while methane formation occurs on iron carbides, C-C coupling reactions are notably more facile, thus underscoring the exceptional activity of iron carbides in FTS and their remarkable selectivity towards olefins.

**Figure 6:**
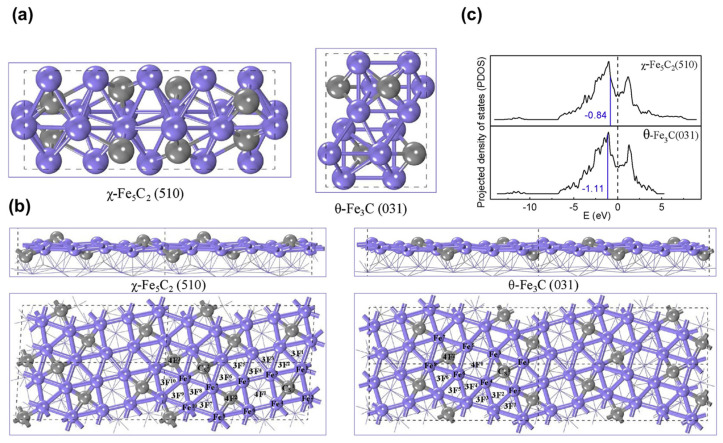
(**a**) Geometric structures of χ-Fe_5_C_2_ and θ-Fe_3_C unit cells. (**b**) Side and top views of χ-Fe_5_C_2_ (510) and θ-Fe_3_C (031) surfaces. (Fe atoms in purple, C atoms in gray). (**c**) Projected density of states (PDOS) of the D-orbitals of the surface Fe atoms for χ-Fe_5_C_2_ (510) and θ-Fe_3_C (031) surfaces. The vertical black dashed lines represent the Fermi level and the vertical blue lines indicate the D-band center. Reprinted with permission from Liu et al. [97]. Copyright 2020 Elsevier. (Fe atoms in purple, C atoms in gray).

**Figure 7:**
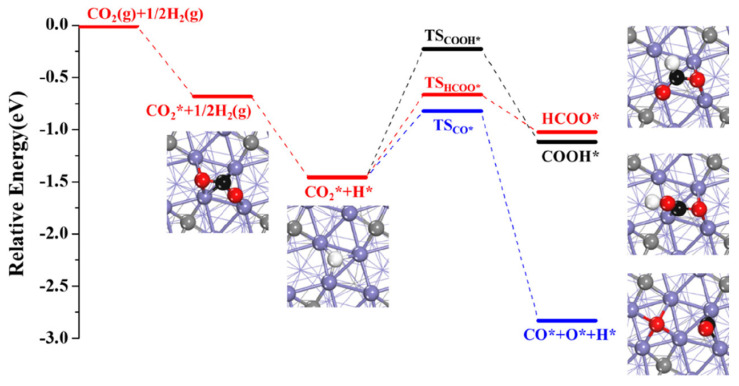
Energy profiles of the three possible pathways for CO_2_ initial conversions over the χ-Fe_5_C_2_(510) surface. (Purple: Fe; black: C of adsorbates; red: O; white: H; and gray: C of χ-Fe_5_C_2_ catalyst). Reprinted with permission from Wang et al. [100]. Copyright 2022 American Chemical Society.

Furthermore, a comparison of CO_2_ conversion pathways on the two carbides have been conducted. Liu et al. [97] investigated the mechanisms of CO_2_ adsorption and activation on thermodynamically stable χ-Fe_5_C_2_ (510) and θ-Fe_3_C (031) surfaces (Figure 6). They explored four primary pathways for CO_2_ activation, including direct dissociation and intermediates involving H, such as COOH*, HCOO*, and CO* + OH* (Figure 5c). The findings suggested that both surfaces exhibited activity for the direct dissociation pathways and were unfavorable for the formation of COOH* [19,77,98]. On χ-Fe_5_C_2_ (510), CO* + OH* could be formed in a single step, while θ-Fe_3_C (031) favored the formation of HCOO* and CO* + OH* intermediates. Additionally, on Fe_5_C_2_ (510), hydrogen displayed distinct behavior across four different adsorption sites (four-fold site, three-fold site, bridge site, and top site). Zhang et al. [99] observed that hydrogen at the three-fold site and four-fold site did not participate in hydrogenation reactions but tended to migrate to another three-fold site or four-fold site, resulting in increased hydrogen coverage on Fe_5_C_2_ (510). Meanwhile, hydrogen at the top site and bridge site engaged in hydrogenation reactions on Fe_5_C_2_ (510). Wang et al. [100] utilized DFT and microkinetic modeling to investigate the mechanism of CO_2_ hydrogenation to hydrocarbons over iron carbide catalysts. The study explored three possible initial conversion pathways of CO_2_ on the χ-Fe_5_C_2_ (510) surface, including CO_2_ dissociation to CO, CO_2_ hydrogenation to COOH*, and HCOO* intermediates (Figure 7). The formation of COOH* is energetically unfavorable, and even if it can be formed on the χ-Fe_5_C_2_ (510) surface, it will dissociate into CO* and OH* species with a potential barrier of 0.72 eV. On the other hand, the formation of the HCOO* intermediate occurs when the surface H* attacks the C atom of CO_2_ to form a C-H bond, and this process is kinetically more favorable than the formation of COOH* via an O-H bond. The energy barrier for CO_2_ dissociation into CO* and O* is 0.64 eV, making it kinetically more favorable compared to COOH* and HCOO*. The calculated results suggest that direct dissociation to CO* and hydrogenation to HCOO* are the two main pathways on the χ-Fe_5_C_2_ (510) surface.

An in-depth understanding of the adsorption and dissociation processes of H_2_O is essential for comprehension of the impact of iron carbide oxidation by H_2_O on the FTS process. Gao et al. [101] employed the GGA-PBE method to investigate the adsorption and dissociation of H_2_O on the Fe_5_C_2_ (010) surface. Their investigation revealed distinct iron-rich and carbon-rich regions on the Fe_5_C_2_ (010) surface, where the adsorption energy results suggested that the iron-rich region is active for H_2_O adsorption and dissociation, while the carbon-rich region remains inert. Notably, H_2_O tends to preferentially adsorb on the top position of surface iron atoms within the iron-rich region. Both thermodynamic and kinetic analyses indicated that H_2_O dissociation in the iron-rich region is energetically favored. Furthermore, thermodynamic analysis elucidated that the catalyst surface continuously adsorbs oxygen atoms in a water environment, with the quantity of oxygen atoms in the iron-rich region contingent on temperature and water content. High temperatures and low H_2_O partial pressures were found to promote catalyst stability. By comparing relevant findings, it can be inferred that the adsorption and dissociation of H_2_O on Fe_5_C_2_ (010) at low coverage closely resemble those observed on Fe (100).

### 3.2. Promoters

#### 3.2.1. Alkali Metals

Alkali metal salts (or oxides) are commonly utilized as electronic promoters in iron-based catalysts, facilitating electron donation and adjustment of the electronic structure on catalyst surfaces. This enhancement augments the adsorption strength of CO_2_ and reduces the energy barrier for olefin desorption. Nevertheless, their specific impact on catalytic properties and mechanisms remains a topic of ongoing debate [102].

**Figure 8:**
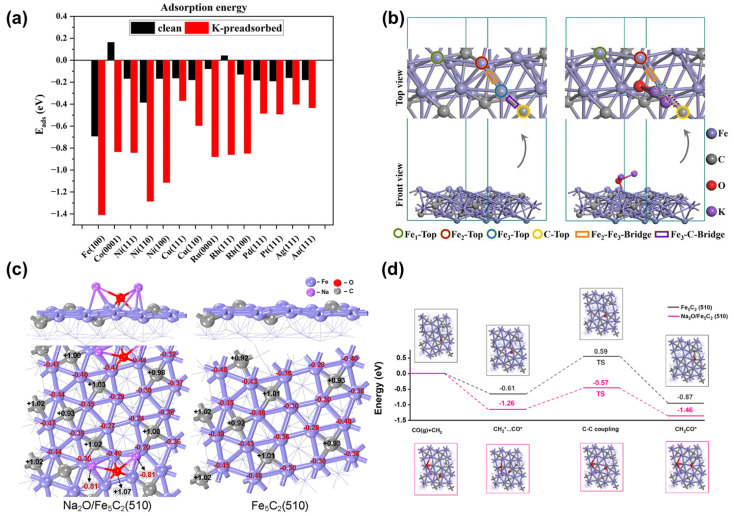
(**a**) CO_2_ adsorption energies on clean and K-preadsorbed transition metals. Reprinted with permission Ma et al. [103]. Copyright 2023 Springer Nature. (**b**) Supercells of clean and K_2_O-adsorbed Fe_5_C_2_ (110). Reprinted with permission Chen et al. [105]. Copyright 2022 Elsevier. (**c**) Electron distribution diagrams of surface atoms on Fe_5_C_2_ (510) and Na_2_O/Fe_5_C_2_ (510) (Fe atoms in purple, C atoms in gray, O atoms in red, and Na atoms in amaranth). Reprinted with permission Liu et al. [106]. Copyright 2021 American Chemical Society. (**d**) Energy profiles of the C–C coupling between CH_2_* and CO* species (CH_2_* +CO* → * + CH_2_CO*) on Fe_5_C_2_ (510) and Na_2_O/Fe_5_C_2_ (510). Na, Fe, C, O, and H are shown in purple, blue, gray, red, and white, respectively. Reprinted with permission Wang et al. [107]. Copyright 2021 American Chemical Society.

The electron interaction between potassium (K) atoms and CO_2_ molecules enhances the adsorption strength of CO_2_, while K_2_O promotes the formation of highly active surfaces. Nie et al. [19] studied the effects of potassium on the adsorption, activation, and dissociation of CO_2_ on the surfaces of Fe (100), Fe_5_C_2_ (510), and Fe_3_O_4_ (111). The research demonstrated that the electronic interactions between CO_2_ molecules and the K-covered surfaces significantly enhance the adsorption and activation of CO_2_ by potassium. The order of adsorption strength follows the order oct-Fe_3_O_4_ (111) > Fe (100) > Fe_5_C_2_ (510). In the presence of potassium, the dissociation barrier of CO_2_ molecules is reduced, favoring CO_2_ dissociation on the Fe (100) and Fe_5_C_2_ (510) surfaces. However, the influence of potassium on the dissociation of CO_2_ on the Fe_3_O_4_ (111) surface appears limited, as a relatively high dissociation barrier persists. Ma et al. [103] examined the adsorption and activation of CO_2_ on clean and K-precovered transition metals. CO_2_ activation on K is primarily governed by electronic and geometric factors. Introduction of potassium significantly enhances the binding strength of CO_2_ and generally reduces activation energy (Figure 8a). This enhancement is mainly attributed to direct electron transfer and dipole–dipole interactions. Huo et al. [104] utilized K_2_O/Fe as a model catalyst and investigated its morphology control effect through both theoretical and experimental approaches. Their findings indicate that the K promoter can modify crystal orientations, favoring the formation of crystals with abundant highly active surfaces. This stabilization effect alters the relative growth rates of crystals in different directions, providing insights for designing catalysts with controllable surface structures. Chen et al. [105] explored the activation of CO_2_ and H_2_ molecules on the surfaces of Fe_5_C_2_ (110) and K_2_O/Fe_5_C_2_ (110), revealing the promoting effect of K_2_O on the hydrogenation of CO_2_ to olefins (Figure 8b). Their research suggests that the adsorption of K_2_O facilitates the direct activation of CO_2_ into CO*, thereby promoting the C-C coupling reaction between CO* and the surface carbon of Fe_5_C_2_. Regarding the activation of H_2_, the K_2_O-adhered Fe_5_C_2_ (110) surface forms Fe-H bonds, which not only lowers the reactivity of H species but also reduces CH_4_ species, preventing the excessive hydrogenation of olefins to saturated alkanes.

Sodium (Na) not only engages in electrostatic interactions similar to potassium (K) but also, Na_2_O significantly alters the surface electronic structure, thereby enhancing olefin selectivity. Mahyuddin et al. [108] investigated the impact of Na and K on the adsorption properties of CO and H_2_S on the Fe (100) surface. Through local density of states and Bader charge analysis, they found that the alkali metal adsorbates reinforce CO adsorption due to the electrostatic interactions between the adsorbates and the molecules. However, they noted a poisoning effect hindering H_2_S adsorption. Liu et al. [106] combined experiments and DFT calculations to systematically study the promoting effect of sodium on CO_2_ hydrogenation over Fe_5_C_2_ (Figure 8c). Through a comparative analysis between Na_2_O/Fe_5_C_2_ (510) and Fe_5_C_2_ (510) models, they elucidated that Na could significantly modify the electronic structure of the Fe_5_C_2_ (510) surface, leading to a reduction in the CO_2_ dissociation barrier from 0.45 eV to 0.08 eV. Furthermore, Na impedes further hydrogenation of CH_2_, enhances C-C coupling, promotes CH_2_ chain growth, lowers methane selectivity, and enhances olefin selectivity. Wang et al. [107] successfully designed a novel multifunctional catalyst integrating Na-doped Fe-based catalyst (Na-Fe@C) and K-doped CuZnAl (K-CuZnAl) for promoting the direct synthesis of ethanol and olefins through CO_2_ hydrogenation. Based on experimental results, they established two surface models, Fe_5_C_2_ (510) and Na_2_O/Fe_5_C_2_ (510), to estimate the C-C coupling barriers (Figure 8d). The computational results are consistent with the changes in ethanol selectivity obtained from multifunctional catalysts with different levels of Na doping. According to Partial Density of States (PDOS) analysis, the low adsorption energy of high-occupied intermediate species is attributable to anti-bonding states on the Na-doped Fe_5_C_2_ (510) surface.

Na binds to the metal oxide MgO, which acts as a catalyst for electron transfer and structural stability. Ahmed et al. [109] designed a dual-functional Na-FeMgOx catalyst and explored the promoting effect of MgO in Fe-based catalysts. Their study revealed that MgO facilitates electron transfer to the Fe-based phase, promoting the reduction of Fe oxides and the creation of oxygen vacancies. Furthermore, MgO enhances the adsorption of CO_2_ and H_2_, thereby facilitating the formation of long-chain hydrocarbons. However, as the reaction progresses, MgO may convert to inactive MgCO_3_, leading to catalyst deactivation and decreased selectivity towards C_5+_ products.

#### 3.2.2. Transition Metals

Transition metals such as zinc (Zn), nickel (Ni), copper (Cu), and manganese (Mn) are commonly incorporated into iron-based catalysts to fine-tune the content and composition of active phases. This adjustment can lead to improved carbon chain elongation and the overall durability of iron-based catalysts.

**Figure 9:**
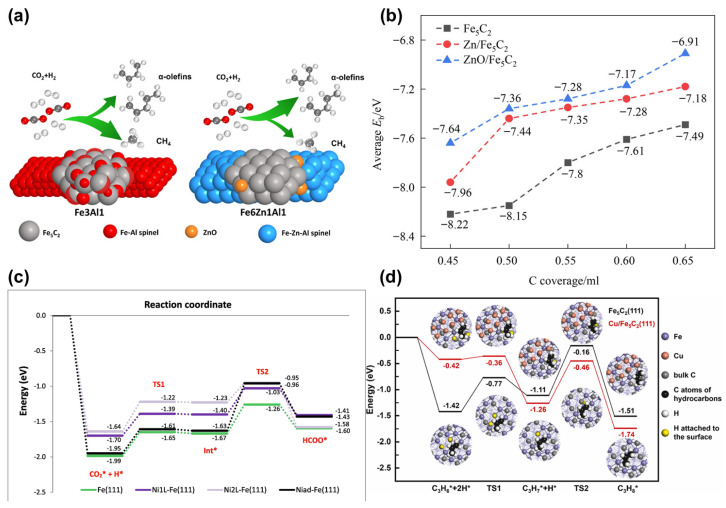
(**a**) Mechanistic diagram of binary Fe–Al and ternary Fe–Zn–Al catalysts for selective synthesis of α-Olefins from CO_2_ hydrogenation. Reprinted with permission from Xu et al. [110]. Copyright 2021 American Chemical Society. (**b**) The average binding energy (E_b_) of isolated carbon atoms on χ-Fe_5_C_2_ (510), Zn/χ-Fe_5_C_2_ (510), and ZnO/χ-Fe_5_C_2_ (510) surfaces at different coverages. Reprinted with permission from Liu et al. [21]. Copyright 2023 Elsevier. (**c**) Energy profiles for HCOO* formation from the MS configurations of CO_2_ on all the surfaces evaluated. Reprinted with permission from Belelli et al. [112]. Copyright 2023 Elsevier. (**d**) Reaction pathways for propene hydrogenation into propane on the Fe_5_C_2_ (111) (black line) and Cu/Fe_5_C_2_ (111) (red line) surfaces. Reprinted with permission from Yang et al. [113]. Copyright 2023 Elsevier.

Zn prevents the re-oxidation of iron carbide and carbon deposition to control surface coverage, ensuring that the phase maintains sufficient dispersion and stability. Xu et al. [110] reported a ternary iron-based catalyst (Fe-Zn-Al) in which Zn was doped into a binary Fe-Al spinel for the direct conversion of CO_2_ into linear α-olefins (Figure 9a). A combination of various in situ characterization methods and DFT calculations indicated that the introduction of Al led to the wrapping of active Fe_5_C_2_ nanoparticles within the Fe-Al spinel, facilitating hydrogenation reactions and inhibiting C-C coupling on the catalyst. The additional introduction of Zn allowed for the redistribution of Al, weakening the strong interactions between Fe_5_C_2_ and the spinel phase and resulting in higher selectivity towards olefins. In a subsequent study, Xu et al. [111] synthesized a series of FeAl catalysts (FeAl350, FeAl450, FeAl600, FeAl750, and FeAl900) at various calcination temperatures for CO_2_ hydrogenation. They analyzed the activity and selectivity of different iron species during the hydrogenation process. Combining experimental and DFT computational approaches, it was found that strong Fe-Al interactions hindered the reduction and carburization of Fe species, resulting in a higher FeO_x_ content. The FeAl350 catalyst, with the highest FeC_x_ content, exhibited the highest CO_2_ conversion rate (48.2%). Lower FeC_x_ content led to decreased activity in CO_2_ hydrogenation to olefins, as observed in the FeAl900 catalyst (36.6%). Therefore, adjusting the composition of active phases offers a promising strategy for the rational design of catalysts for CO_2_ hydrogenation to olefins. The study by Liu et al. [21] investigates the role of Zn-promoted iron-based catalysts in the direct hydrogenation of CO_2_ to produce olefins. The research reveals that in the presence of Zn, the active phase Fe_5_C_2_ (510) can prevent further oxidation under reaction conditions and the deposition of carbon on the catalyst surface (Figure 9b), leading to enhanced long-term stability. Zn facilitates the adsorption of surface oxygen atoms and promotes the desorption of H_2_O and H_2_O during hydrogenation, reducing the likelihood of surface carbides being oxidized. The study speculates that in terms of stabilizing iron carbides, the effect of ZnO is more significant compared to standalone Zn.

The introduction of Ni impedes the adsorption of CO_2_ on Fe (111). Belelli et al. [112] examined the adsorption and dissociation of CO_2_ on the Fe (111) surface and three different Ni-Fe (111) alloy surfaces, focusing primarily on the most stable (MS) and most active (MA) modes (Figure 9c). Overall, the presence of nickel inhibits the adsorption of CO_2_ compared to the Fe (111) surface. The study proposed two different mechanisms for CO_2_ dissociation: one starting from the MS mode, directly producing CO and O through dissociation; the other involving a two-step reaction, first migrating from the MS mode to the MA mode, followed by dissociation in the MA mode. From a kinetic perspective, the pure Fe (111) surface is highly favorable for hydrogenation reactions, while the presence of nickel increases the hydrogenation barrier in all cases, leading to the formation of the HCOO* intermediate.

Cu modifiers demonstrate a comparable effect to Zn in promoting the reduction and carburation of iron-based catalysts, thereby favoring the formation of Fe_3_O_4_ and Fe_5_C_2_ phases. However, strong interactions between Cu and iron species impede CO adsorption, unfavorably influencing the formation of CO intermediates. Yang et al. [113] conducted a combination of various characterization techniques and DFT calculations to investigate the promoting effects of Zn, Cu, and Mn on Fe-based catalysts for the catalytic hydrogenation of CO_2_ to produce olefins. FeZn-Na exhibited excellent activity, displaying the highest overall selectivity towards olefins. Based on experimental characterization, models of the Fe_5_C_2_ (111), Cu/Fe_5_C_2_ (111), and ZnO/Fe_5_C_2_ (111) interfaces were constructed. In the transition from C_3_H_6_ through two hydrogenation steps to C_3_H_8_, a notable increase in the energy barrier in the second step was observed, suggesting that the Cu/Fe_5_C_2_ (111) (Figure 9d). However, calculations indicated that further adsorption and hydrogenation of olefins on ZnO/Fe_5_C_2_ (111) are unfavorable. 

#### 3.2.3. Synergistic Effect

**Figure 10:**
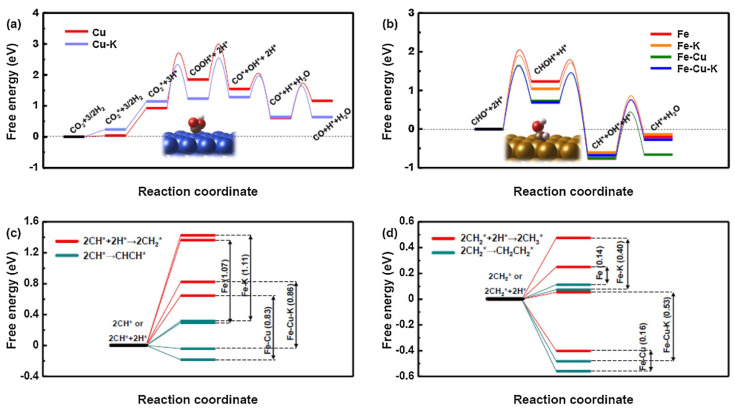
Relative energy diagrams for (**a**) the RWGS mechanism over Cu and CuK surfaces; (**b**) the CH* formation mechanism over Fe, FeK, Fe-Cu, and Fe-Cu-K surfaces. The effect of K on the chain growth mechanism from (**c**) CH* and (**d**) CH_2_* monomer. T = 300 °C and P = 2.5 MPa. Reprinted with permission from Hwang et al. [114]. Copyright 2019 Elsevier.

Exploiting the inherent advantages of incorporating alkali metals to catalyze active phase formation and enhancing the structural robustness of iron-based catalysts through the addition of transition metals, the concurrent integration of alkali and transition metals represents a strategic approach to amplify catalytic efficacy in CO_2_ hydrogenation. Hwang et al. [114] developed a Fe-Cu-K catalyst for investigating the hydrogenation of CO_2_ (Figure 10). Experimental findings demonstrated that the yield of C_5+_ products was 18.1%, which is 1.4 times higher than that of the FeK catalyst (12.8%) and 7.8 times higher than that of the Fe-Cu catalyst (2.3%). Experimental characterization confirmed the incorporation of copper into the lattice of iron in the presence of potassium. The addition of K ensured the activity of the FeCu alloy phase during the reaction. Electron structure calculations revealed that the Fe-Cu surface promotes CO_2_ hydrogenation by lowering the energy required for deoxygenation. Regardless of the reaction pathway, the addition of K increased the binding energy of carbon and decreased the binding energy of hydrogen. The study speculated that surface modification might increase the surface carbon density, explaining the high selectivity of the K-promoted catalyst for the formation of long-chain hydrocarbons. Subsequently, Hwang et al. [115] synthesized a novel Fe-Co alloy catalyst derived from N-coordinated Co single-atom carbon (FeK/Co-NC) and applied this catalyst system to CO_2_ hydrogenation. Due to the Co-NC support, Co atoms were efficiently supplied to Fe nanoparticles, inducing the effective formation of Fe-Co alloy. DFT calculations indicated that Fe-Co mixed oxides accelerate oxygen removal in the RWGS reaction, while Fe-Co mixed carbides promote chain growth and suppress methane formation in FTS. This innovative catalyst design not only showcases the potential of Fe-Co alloys for catalytic applications but also highlights the synergy between different components in achieving enhanced catalytic performance in CO_2_ hydrogenation.

## 4. Conclusions and Outlook

In conclusion, this review provides a concise overview of recent theoretical advancements in iron-based catalysts for CO_2_ hydrogenation, employing DFT simulations and microkinetic models. Various types of iron-based catalysts, including metallic iron, iron oxides, iron carbides, and other derivatives, have systematically been synthesized to understand the mechanisms underlying CO_2_ hydrogenation. Notably, alkali metals have been shown to significantly enhance CO_2_ adsorption and stabilize active phases within iron-based catalysts. Additionally, the incorporation of doped transition metals contributes to the development of versatile catalysts with multifunctional capabilities. The catalytic functionalities of each metal species enable distinct transformations of target molecules. The synergistic integration of alkali metals and transition metals allows precise adjustments of key attributes governing the CO_2_ hydrogenation process. These findings highlight the efficacy of the bottom-up catalyst design strategy, facilitating the production of high-performance iron-based catalysts for CO_2_ hydrogenation.

Despite the remarkable achievements of computational methods in identifying active sites and elucidating structure–activity relationships in Fe-based catalysts for CO_2_ hydrogenation, their practical applicability has been confined to specific spatial and temporal scales for which they were developed. Artificial intelligence (AI) and machine learning (ML) present promising avenues for enhancing theoretical descriptions across various time and length scales. Integrating density functional theory (DFT)-based materials databases with ML approaches can facilitate the transformation of data into valuable insights, thereby accelerating the discovery of catalyst candidates and enriching our understanding of underlying catalytic mechanisms. Although in its early stages with many underexplored research opportunities, ML techniques have demonstrated successful applications in various solid heterogeneous catalysis in recent years [116,117,118]. With the ongoing increase in applied research, we anticipate that machine learning methods will emerge as pivotal computational tools for advancing the development of high-performance catalysts for CO_2_ hydrogenation in the near future.

## Figures and Tables

**Table 1:** Microkinetic analysis of the redox and association reaction steps in the reverse water–gas shift reaction. Reprinted with permission from Pahija et al. [12]. Copyright 2022 American Chemical Society.

Reaction Steps	Redox Pathway	COOH Pathway	HCOO Pathway
CO + * ⇆ CO*	CO_2_ + H_2_	CO_2_ + H_2_	CO_2_ + H_2_
H_2_ + 2* ⇆ 2H*	CO_2_* + 2H*	COOH_trans_* + H*	HCOO* + H*
H_2_O + * ⇆ H_2_O*	CO* + O* + 2H*	COOH_cis_* + H*	HCO* + O* + H*
H_2_O + * ⇆ H* + OH*	CO* + OH* + H*	CO* + OH* + H*	CO* + O* + 2H*
CO_2_* ⇆ CO_2_ + *	CO* + H_2_O*	CO* + H_2_O*	CO* + OH* + H*
OH + * ⇆ O* + H*	CO* + H_2_O	CO* + H_2_O	CO* + H_2_O*
OH* + OH* ⇆ H_2_O* + O*	CO + H_2_O	CO + H_2_O	CO* + H_2_O
CO* + O* ⇆ CO_2_* + *			CO + H_2_O
CO* + OH* ⇆ COOH_cis_* + *			
COOH_trans_* + * ⇆ CO_2_* + H*			
COOH_trans_* + OH* ⇆ CO_2_* + H_2_O			
CO* + OH* ⇆ HCOO* + *			
CO_2_* + H_2_O* ⇆ HCOO* + OH*			
OH* + OH* ⇆ H_2_O* + O*			
COOH* + * ⇆ HCOOH**			
COOH_cis_* ⇆ COOH_trans_*			

## Data Availability

The data presented in this study are available in this article.
